# Falling giants and the rise of gene editing: ethics, private interests and the public good

**DOI:** 10.1186/s40246-017-0116-4

**Published:** 2017-08-29

**Authors:** Benjamin Capps, Ruth Chadwick, Yann Joly, John J. Mulvihill, Tamra Lysaght, Hub Zwart

**Affiliations:** 1 Department of Bioethics, Faculty of Medicine, Dalhousie University, Halifax, Canada; 2 School of Law, University of Manchester, Manchester, UK; 3 Department of Human Genetics, Centre of Genomics and Policy, McGill University, Québec, Canada; 4 Department of Pediatrics, University of Oklahoma Health Sciences Center, Oklahoma, USA; 5 Centre for Biomedical Ethics, Yong Loo Lin School of Medicine, National University of Singapore, Singapore, Singapore; 6 Faculty of Science, Department of Philosophy and Science Studies, Radboud University Nijmegen, Nijmegen, The Netherlands

**Keywords:** Public good, Public interest, CRISPR, Solidarity, Benefit sharing, Ethics in genomic research, Human genome project, Biobank

## Abstract

This paper considers the tensions created in genomic research by public and private for-profit ideals. Our intent is to strengthen the public good at a time when doing science is strongly motivated by market possibilities and opportunities. Focusing on the emergence of gene editing, and in particular CRISPR, we consider how commercialisation encourages hype and hope—a sense that only promise and idealism can achieve progress. At this rate, genomic research reinforces structures that promote, above all else, private interests, but that may attenuate conditions for the public good of science. In the first part, we situate genomics using the aphorism that ‘on the shoulders of giants we see farther’; these giants are infrastructures and research cultures rather than individual ‘heroes’ of science. In this respect, private initiatives are not the only pivot for successful discovery, and indeed, fascination in those could impinge upon the fundamental role of public-supported discovery. To redress these circumstances, we define the extent to which progress presupposes research strategies that are for the public good. In the second part, we use a ‘falling giant’ narrative to illustrate the risks of over-indulging for-profit initiatives. We therefore offer a counterpoint to commercialised science, using three identifiable ‘giants’—scientists, publics and cultures—to illustrate how the public good contributes to genomic discovery.

## Introduction

Cutting-edge bioscience is a public good: in addition to economic benefits, it can generate social value in healthcare, agriculture and industry. Sometimes, however, preoccupation with a pecuniary imperative encourages ‘hype and hope’: predictions that beget idealism and claims which promise too much. Hyperbole has been a feature of genomics since its inception, and high hopes continue to shape perceptions of private interests and the public good. In this paper, we present an egalitarian-type response to the entrenchment of commercialisation in genomics research. Using the concept of genomic solidarity, we endorse undertaking research for the public good and question the current commercial speculation in genomics.

From the Human Genome Project (HGP) [1] as the flagship project of the ‘Genomic Era’ [2] up to the new wave of post-genomics research, there has been an over-arching narrative about the value of high-profile discoveries. Most recently, this has been highlighted by gene editing—a platform of converging scientific expertise organised around similar methods—and specifically, CRISPR-Cas9. As we discuss later, these discoveries are often promoted as the exclusive achievement of processes of commercialisation. This gratification bias, which creates pathways for exclusion and validates outlandish (and sometimes unjustified) rewards for innovators, is at least partly responsible for the devaluing of the public infrastructure. On closer inspection of the sophisticated pathways of scientific discovery, it becomes clear that in various ways, the quest for significant returns potentially jeopardises the ways that the public good contributes to the production and implementation of scientific knowledge. It is, therefore, essential that responsible research in genomics and post-genomics include the management of promises (or ‘promisomics’) [3] which we argue requires the reification of the public good. We contend that society-driven research anchored in the public good should be acknowledged as essential to progress. Refocusing upon the public good could, to some degree, challenge the culture of hype and hope [4].

The paper is structured in two parts. In the first part, we situate genomics within a ‘giants’ narrative. In making our case for the public good, we draw on the work of Robert Merton, who recounts the Newtonian idea of giants in science upon whose shoulders others stand [5]. Applying this to genomics, we argue that the giants are collective infrastructures and broad research cultures. With these in mind, we then offer a challenge to market ideologies as the pivot for successful discovery by emphasising the extent to which progress *presupposes* research strategies that are ‘for the public good’.

In the second part, we build a ‘falling giant’ narrative to illustrate the risks of over-indulging for-profit initiatives that come about because of the predominant ideology that is imposed upon research. That trend has devalued the public good. Thus, we offer a counterpoint to marketisation using three ‘giants’—scientists, publics and cultures—to illustrate how the public good contributes to genomic discovery.

## Giants and genomic technologies

CRISPR-Cas9 (hereafter CRISPR) is the latest highly prized biotechnology. It is a gene editing tool developed from bacterial adaptive immunity, based on Clustered Regularly Interspaced Short Palindromic Repeats and ‘**C**RISPR-associated’ enzymes. It is a precise, rapid and cheap tool for editing DNA that by far outperforms previous genetic engineering capabilities; it has become essential in laboratories across the globe. Like the HGP before, CRISPR promises to revolutionise genetics and genomics as a quantum advance, and much like the sequencers who laid the groundwork for next-generation technologies, it will allow superior analytics to become available to diverse laboratories [6].^1^ The emergent gene editing platform is a switch from the slow but widely available genetic engineering tools of yesterday, to new, sharp and shiny ones.

Understanding CRISPR’s place in scientific creativity and its implications for society can suggest ways in which technologies are defined by vested interests, policy goals and public imaginations. In the *emerging landscape* of gene editing technology, a number of themes are re-emerging from past innovations. One of these themes is the anticipation that technology brings vast clinical benefits. Before gene editing, the promise of stem cell science predicted sudden and immediate prospects—a technology that still envisages immense progress in areas like regenerative medicine but has yet to meet expectations. Of ethical concern is the repeated use of hype and hope to attract funding, promote more permissive regulations and mislead vulnerable patients [7]. We do not see this simply as malicious action by greedy scientists or institutions promoting their investments (but witness the recent court battles between scientists and institutions in respect to the CRISPR patents [8]), nor do we dismiss it as the work of media hunting for headlines. Rather, it is a feature of the profit-driven constellation whose basic premises we question—their ‘catallactic bias’ [9]^2^ towards promoting markets as podiums for progress without also questioning their unfairness and their failures, as well as their accomplishments.

A second theme is access to technologies [8]. We may more generally question the pervasive idea that profit is not an afterthought to doing worthwhile science, but the *raison d’être*. While patents are important in biosciences, at least according to the socio-economic argument that they stimulate innovation and investment, critics question the effectiveness (and desirability) of monopolies as incentive models for innovation [10]. To this end, whoever successfully receives the rights to CRISPR could assert to a large extent the still unspecified terms under which that technology is adopted in laboratories in clinical, animal and agricultural areas [11]. The consequences of these legal trials could redefine traditional *genetic engineering*—that has become an inclusive platform over the years—in terms of the exclusive context of modernistic *gene editing*. Critically, patents may also encourage the kinds of unreasonable dominance that elevates inventors and privileges investors, while subordinating public goods. The current landscape of marketisation as means to discovery and value, might push valuations of CRISPR-based therapeutics into the stratosphere of reasonable cost in order to satiate returns. There, they become out of reach of most including insured patients and those dependent on national health systems. In such circumstances, only the wealthiest can be optimistic of benefiting from CRISPR technologies.

However, rather than focusing on legal and clinical access conundrums, we want to recast this debate by using Merton’s narrative about the words famously uttered by Isaac Newton: *Without the giants we would see nothing; on their shoulders we see farther*.^3^ The first giants are the individual innovators and inventors and, in this respect, Newton’s well-known aphorism is an expression for the dependencies of scientific researchers on predecessors. In other words, however original the present endeavour, its success can be traced back to many prior discoveries. The development of CRISPR involved many incremental steps, including the discovery of DNA itself and many contributions since [12, 13].

Second, there are the giants in contemporary biomedical research. Scientists increasingly rely on vast networks and infrastructures, such as big international research consortia, big machines and big data. It is here that one finds further significance in the aphorism in respect to the biases of collaboration (who works with whom and why), particularly in a culture in which being first brings global fame and (not just monetary) fortune [14].

Third among the giants is the enormous influence of culture, pegged to the zeitgeist of any particular time; these might be categorised as neoliberalism, the Anthropocene, populism, post-truth and so on. Gaining cultural ascendency is significant for scientific discovery: it can determine what, where and by whom science is done, and who is acknowledged, compensated and rewarded. In these times, arguably, science is dominated by neoliberalism, and that involves planning scientific research ultimately to translate discoveries into consumer products and industrial technology; progress, in this respect, is possible only because of the ascendency of corporations, competition and ‘degovernmentalization’ [15]; innovations and discoveries are celebrated primarily because of their exceptional contributions to the vast biomedical-market. As a result, the current CRISPR debate is dominated by the clinical prospects rather than the undoubted contributions it will make in many other areas such as animal and agricultural engineering. However, it is our conjecture that within all three giants, the real contributions of the public good are distorted to make the case for marketisation. To understand how this came about, we need to go back to the ideologies that grounded the genomic revolution.

### Genomics and emerging giants

In 2010, the journal *Nature* asked whether the ‘genomic revolution’ had arrived. Contributors to the issue included the key architects of the HGP, Francis Collins [16] and Craig Venter [17], whose answers, and those of other contributors, were essentially ‘Not yet’. The reference human genome dramatically changed the capabilities of genomic research, yet so far (in 2017), the benefits for individuals and society have been limited. There remain to date three grand challenges in genomics: genomics to biology (elucidating the structure and function of genomes), genomics to health (translating genome-based knowledge into health benefits) and genomics to society (promoting the use of genomics to maximise benefits and minimise harms in populations) [2]. The revolution is progressing more slowly than many first envisioned; in particular, there is still some way to go in the translation of genomic science into widespread clinical applications. It is difficult to pinpoint any one single reason for this [18], but perhaps it is the right time to consider conceivable flaws in the ideologies that inform the industry-research complex responsible for undertaking genomic sciences.

The HGP was a moment of high visibility for science that attracted vast public financing and private entrepreneurship^4^; now, standing on the shoulders of *this* giant, we can appreciate discoveries such as CRISPR. Maintaining momentum in genomics has become the hard sell to investors and funders, both public and private, so that waves of hype (and some hope) continue to fluctuate [3]. While technologies become more effective, our dexterity in managing expectations hardly seems to improve at all; for genomics, prospects are being transferred to new initiatives, such as personal and precision genomics [19], and now, gene editing can be added to that list.

Reflecting on the HGP, Maynard Olson writes:

There are two stories of the Human Genome Project. One describes a century of scientific progress that began with the rediscovery of Mendel’s laws in 1900 and ended in a frenzy of genomic sequencing. The other is a story about contemporary societal values—particularly those that framed the project’s endgame and continue to shape public perceptions towards this defining event in time ([20], p. 931).

The first story alludes to the many giants that enabled progress in genetics and genomics—all of which surely contributed, in various ways, to the post-genomic era; that must include many other confluent technologies such as computing and data storage. The idea to sequence the human genome, then, was as much about historical socio-political events as about the technological feasibility that would lead to an opportune ‘time to sequence’ [21].

The second story is about translating genomics into society. The HGP was characterised by some as a race between two competing parties—the International Human Genome Sequencing Consortium (IHGSC) [22] and Celera Genomics [23]. It is a story that is multi-layered, involving partisan politics and indiscriminating press coverage documented in the public-private competition between the ‘players’ [20]. At the time, two key players claimed a special connection to the public interest or good—the public project of IHGSC wanted to publish their sequences so that it was freely and therefore widely accessible; Celera argued that it could get the job done more quickly and save countless human lives by using intellectual property to generate exclusive rights and revenue from the human genome [20]. Taking liberty to distill that rivalry to its most basic point, two ideologies surface: on the one hand, Venter and Celera’s interest in sequencing the human genome was billed as a way to accelerate the laboured efforts of the public initiative. On the other hand, Collins, praising the public investment as ‘arguably one of the more impressive success stories… of all time’, recognised the implications if the Consortium ‘dropped the ball’ ([24], p. 60, 80); the only way to assure unrestricted access to the sequence was to continue with the public project, perhaps in partnership with other private entities [24]. Thus, it was either a story of mavericks challenging the slow-witted establishment or a lament about how private interests seemed about to *capture* public goods [25].^5^ These competing ideologies persist in bioscience today, often because of promises and pitfalls of scientific research are created, sustained and leveraged via ethical and social norms expressed by the leaders in the field. These opinions echo within complex social and political networks and are sustained by immense public and private infrastructures.

### The public good

The response of Collins to Celera’s strategy was to reaffirm the significance of the public good. What, then, is meant by the public good?

The Human Genome Organisation (HUGO) has a long tradition of advocacy for ‘benefit sharing’ to realise societal *as well as* economic opportunities [26]. In a HUGO statement from 2000, it was stated:

A benefit is a good that contributes to the well-being of an individual and/or a given community. ... Thus, a benefit is not identical with profit in the monetary or economic sense. Determining a benefit depends on needs, values, priorities and cultural expectations… The HUGO Ethics Committee recommends … that all humanity share in, and have access to, the benefits of genetic research [27].

HUGO’s statement, we believe, reflects the public-private intellectual climate of that time.^6^ At the outset of the HGP, it was proposed, and then codified in the 1997 Bermuda Principles, that human DNA sequences ‘should be freely available and in the public domain in order to encourage research and development and to maximise its benefit to society.’

It was agreed that these principles should apply for all human genomic sequence generated by large-scale sequencing centres, funded for *the public good*, in order to prevent such centres establishing a privileged position in the exploitation and control of human sequence information [our emphasis.] [28]

The HUGO Committee on Ethics, Law and Society has more recently stated that ‘genomic solidarity’ ideally supports collaborations between individuals, communities and populations, with research communities and industry [29].^7^ Significantly, benefit sharing and genomic solidarity work together through an idea of the public good [30]. In the most rigorous terms, benefit sharing suggests that research must be preceded by engagement with all stakeholders rather than allowing for exclusion and domination and, consequently, disunity. Relatedly, a notion of solidarity requires collective agreement upon common ends to be achieved and how to do so, and thereby, differentiates between public goods and public bads. A public good is valued distributively, i.e., to each and every person that value is secured through equality of rights. In rights talk, that idea recalls the right to claim a good (such as food, water or shelter) and affirms the justified protection of persons’ important interests (to claim a right is to claim access, protection or provision of a good). That claim is not limited to the goods that are traded but includes all goods that establish a basic level of healthy living and contributes to opportunities, within egalitarian societies. Public bads do the opposite: they exist in a way that affect people distributively (such as pollution spewing into a river from a factory upstream of a village) and are expressed in terms of those affected having their rights infringed. Within a solidary framework, the scope for public goods to do good and public bads to do the opposite is understood; institutions and cultures thereby adapt to priorities that most likely support public goods. In the context of genomics, the public good means that everyone is entitled to access to the fruits of research because that meets HUGO’s ethical conditions for benefit sharing and solidarity.

The idea of genomic solidarity is likely to be challenged, for it confronts the engrained idea of public goods as something which hinders the benefits of economies of exclusion and rivalry. In that classical estimation, goods are ‘public’ depending on whether private investment has any interest in them; in other words, if a good is profitable, then it is economically wasteful to consider it a public one. This illustrates what Samuelson originally called *collective consumption goods* (what later became known as ‘public goods’) [31]. It leads to a particular view of goods that can be applied to the human genome: in the course of human history, every human being, living or dead, has been part of the genome’s conception (for instance, by adding variants) and contributed to its continuation. Although it is our legacy, no one person has written the chapters, and sequencers are now ‘reading’ the book and genomists are ‘translating’ it. This process of curiosity, understanding and innovation converts the genome from a status of *public legacy* to one of *value* (it is now a chapter or verse that deserves a price), and ownership becomes a significant factor in that conversion. In short, particles of, or even the entirety of the genome, have become someone’s property [26].

And now, gene editing is likely to extend the interest in ownership of DNA in the same way that economics shaped the claims of ownership of other human materials [32]. These rewritten or novel sequences will exist outside of any normal or representative human genome. Thus, it is more likely than ever that human genes will become commodities [33], and society will have to decide how tolerable such claims are to be in light of the alleged benefits of a flourishing gene market. It is in this context that we find HUGO’s benefit sharing model and genomic solidarity as a challenge to uncritical characterisations of human genomic commodities. In the next section, we use the narrative of giants to explain the public’s role in genomics, the pitfalls of profit-driven science, and to thereby strengthen the conception of the public good.

## Giants and the public good

How do CRISPR and other gene editing tools become an opportunity for the public good? We now explore in greater detail the ‘giants’ metaphor; a term which not only means ‘greater than normal’ but also refers to ways that people who are exceptional in talents and abilities contribute to ideal conditions for discovery. There are three giants: (1) individuals with great creativity and insight involved in the development of the technology (not just the inventors but also the policy makers, politicians and administrators who will create the regulatory conditions in which gene editing occurs); (2) institutions of great size and reach, where research is housed and applications transpire; and (3) the prevailing zeitgeist, namely the cultures that exert influence in this area of research.

### Individuals as giants

The prevailing social narrative of CRISPR concerns the first of our giants: inventors pitted against one another in conjunction with their lawyers and administrators [34]. As a result of this perspective, there is a tendency to think about cutting-edge technologies only in terms of economics, thus venerating scientists for their endeavours within systems that primarily promote profit [35].

However, from the *observation* that clustered repeats might be significant, to CRISPR’s sensational harnessing and refining, it involved, as with nearly all other discoveries, many scientists, working for many years on many topics [36]; CRISPR’s discovery, therefore, is contentious in respect to the ‘giants’ metaphor. On the one hand, a legal narrative prompts us to focus excessively on isolated contributions attributable to individuals; on the other hand, that approach disavows the extent to which novelty builds on vast networks of knowledge and technology that are already in place. This understanding of discovery is also relevant in respect to technology’s translation into applications and useful products [36]. In this respect, we might ask whether the gene editing platform should be a public resource by acknowledging multiple contributions.

Our premise is that the links of discovery are much wider that is currently appreciated by the legal narrative. Rather than standing in isolation, scientists, their affiliates and institutions rely on publics who volunteer their time, bodies and experiences for clinical trials, become patient-participants in research through their giving of data and tissue samples, and have interests by way of meeting their tax obligations (that are spent on industry partnerships and subsidies). Science, therefore, consumes enormous amounts of public time and resources; its progress is felt through the flow of capital, user products, and necessary oversight and regulation. It is because of these factors that scientists are accountable to publics: the public good, therefore, refocuses progress upon what the public needs, or expects, from investments in bioscience. These arguments become more pressing when the technology is as significant as potentially gene editing is. If science is answerable to the public, then there might be an expectation that there are good reasons for commodification. In that case, arguments for the exclusivity for CRISPR might be contentious because of the public interest in public goods and the ways in which markets cause mischief in this respect: the patterns of hype and hope and limited access contribute in ways that are public bads. The public good requires a revaluation of progress so that science justifies investment and rewards, by maximising social progress via promoting pathways in which better medicines lead to better health, and those benefits are reasonably accessible. For instance, perhaps by acknowledging the interdependent paths of thought and discovery, we would become more prudent when rewarding serendipitous discoveries, and, moreover, question industries that often require secrecy and delay dissemination [14]. In these respects, benefit sharing and genomic solidarity acknowledge that discoveries happen, not just because society venerates and rewards innovators, but because their discoveries stand on the shoulders of those that contribute to worthwhile aspects of society.

### Institutions as giants

There are case examples that can usefully show how marketisation effects social progress. We have already seen how the architects of the IHGSC believed approaching the project as a public good was the most effective way of deciphering the human genome and making sure it reached as many as possible. Their efforts are unambiguous in underlining the importance of the capacity for public innovation, and yet, in the hubbub of entrepreneurship, the public contribution is easily overlooked. In fact, because of the HGP, individual innovators stand to benefit from these kinds of gigantic and collective knowledge-producing institutions. Thus, we might think of public research as a broad partnership in which information is shared between institutions, researchers, participants and publics, and this framework signifies the importance of the public good in biosciences.

Examining CRISPR as a broad social phenomenon draws attention to the kinds of institutions that contributed: education (high schools, universities), research and training (research facilities and supervision, as well as public funders), and security and stability (from sophisticated enabling infrastructures up to legal systems). These signify the public infrastructures’ role in innovation. Pierre Teilhard de Chardin once referred to these giant, global, intelligent networks as the ‘noosphere’ (derived from the Greek term νοῦς: i.e. ‘mind’ or ‘intellect’) [37]: the worldwide network of research facilities, discourses, devices, circuits and repositories. He describes a collective and distributed web of collaborators, working together in order to co-create the technologies and insights needed to address global challenges. In other words, collaboration and technical and resource dependency is necessary, and individual achievement is only possible because of these giant techno-scientific networks. It is apt, then, to recall these giants as part of the gene editing narrative about the public good, benefit sharing and genomic solidarity.

When we shift our focus from the innovators to the research participants, for instance, one perhaps recognises the importance of other active and ‘passive’ contributors to science. In this respect, there have been some notable developments since the outset of the Genomic Era, especially under the moniker of *big data*, which segues logically from the vastness of the human genome. Big data applies to the creation of extremely large data sets for computational analysis to generate value [38]; these data are sourced from vast, indiscriminate methods of trawling random information for patterns and opportunities. Others create data within the public commons, namely, a data repository or resource that is ‘of the people’ who voluntarily contribute. In respect to the latter, biobanks have become significant in terms of activating public collaborations in ways that are characterised as ‘for the public good’ [25]. This public good distinction has considerable impact on governance and the norms that define collection methods and processes to use resources. A strong sense of the public good contributes to ‘open science’; conversely, institutions that are pursuing big data for commercial reasons often consolidate and conceal their collections. The latter are the traditional giants of private enterprise, such as the pharmaceutical industry. Their practices for accumulating and sharing these data are much different from the aforementioned public good practices, instead using private business models rather than public engagement to appropriate and withhold data [39]. Sometimes, that business acumen amounts to capture.^5^

An example of public good capture is illustrated by the Icelandic deCODE health sector database (*qua* biobank). The rise and fall of that biobank is a complicated story of political and scientific intrigue that has been widely documented [40]. In essence, proponents for the deCODE biobank claimed there was a public good in aggregating health records to be used by affiliates of the biobank and those purchasing licences. deCODE had to make the biobank attractive to venture capitalists; to do so, they realised that ownership of the data would be necessary. That business strategy was defended by arguing for a public interest for economic growth and national revitalisation (i.e. investment in scientific infrastructure). Many of the data were amassed legally (after a much debated and enacted law), but without having to obtain the express consent of individuals in Iceland (who could only opt out).

That strategy was successfully challenged in court [41], and this proved ultimately a pivotal moment that exposed the inadequacy of deCODE’s ‘public good-public interest’ rhetoric. From the outset, the argument about the public good was doubted by many in the scientific and medical communities [40]. In defence of the strategy, ‘The theme of solidarity, through the idea that deCODE could help keep families together, was invoked to outweigh abstract notions of autonomy, patient–doctor confidentiality, and erosion of scientific integrity’ ([40], p. 89). What the ‘public good’ really meant to deCODE was the embrace of ‘naive scientific hype, commercial dominance, and the privatization of common cultural and scientific resources’ ([40], p. 100). During the days of the HGP, Celera used similar rhetoric about the ‘importance of this information to the entire biomedical research community’; ([20], p. 934) but had no intention of depositing its sequence data into the public GenBank database. The company still intended to restrict public access to their sequence, suggesting that, as Collins’ predicted, (paraphrasing) perhaps it is not a sound market strategy to be giving data away for free ([20], p. 935) (also see [24]).

Why is it important to challenge private data acquisition? Firstly, Collins argued that the fruits of the HGP should be kept in the public realm because he believed in facilitating access as widely as possible: the bottom line was progress through collaboration, rather than progress by the bottom dollar. He felt discovery would come from collaboration, and not privatisation and capture. It is worth pointing out in light of the assumption that public-based research is antiquated, that the public IHGSC sequencers not only competed on time and under budget but also published a more comprehensive genome (Celera’s speed had come with gaps in its sequence that had to be filled using the publicly available data) [1], and, most of all, that success ensured that the human genome remained in the public sphere. Secondly, the public good model means that *all* researchers—not just those allied to the IHGSC, Celera, deCODE affiliates or those willing (or able) to afford licencing costs—have access. That becomes an inclusive path to progress rather than the narrow trajectory of consumerism: it opens up exploration beyond profit motivations. Doing so does not morally shut any doors to profit: venture capitalists would still be welcome and encouraged to develop products and derivative technologies, but without being allowed a monopoly on the tools or data.

Consider an exemplar of this model: the UK Biobank. Its participants are not paid, and they receive few direct benefits. Research is not prioritised simply because it likely leads to profit, but it must contribute to the public good﻿. Half a million people voluntarily and enthusiastically took part. Why? Perhaps they understood the purpose of the biobank to be about the advantages of creating a sustainable public resource, and endorsed its intention to provide inclusive access *for the good of all*. In the case of UK Biobank, the Ethics and Governance Council acts as a ‘steward’ of participants’ data and samples, and therefore takes a direct responsibility for their interests [30]. This role is only possible because of a governance framework that incorporates participants’ rights as conceived by the public interest and public good, and creates a broad steering role for participants through a vision that is informed by ethics rather than business. Even so, UK Biobank does recognise ‘reasonable’ patents, which refers to inventions that are ‘not used to restrict health-related research and/or access to healthcare anywhere in the world.’^8^ Compare that to deCODE’s strategy to exploit the enthusiasm of the Icelandic people or Celera’s mission. Both had begun with overt economic ideals to capture the public good.

The examples of IHGSC and UK Biobank, we believe, counter the presumptive folly of public ineptitude, and indeed illustrate ethically and economically reasonable arguments to support public goods [42].

### The culture of giants

The US National Research Council saw the opportunity for creating a framework to create, manage and coordinate access to the vast amounts of information generated from genomics research, but did not state how this was to happen. Instead, they urged the key players, agencies and institutions to grow into their roles to avoid stifling innovation and adaptation [43]. However, developments in biobanking, where there are clear signs that the public good can be enhanced for inclusive benefit [44], and the tempering of patent claims over human genes,^9^ suggest the possibilities for securing genomics for the public good. Capture, in this respect, has a more general danger: ‘There is a significant risk that if certain commercial deals are struck or if public access is somehow limited, there may be a real or perceived sense in which managers have reneged on an implied promise to advance the “public good”’ ([45], p. 449). The problem resides in ‘the corporate skew of the research agenda’ ([45], p. 448). Capture is also an issue of trustworthiness. Holding institutions to be trustworthy is far more significant for those in the public sphere, where publics expect their interests to be respected (such as privacy), than those clear about the priorities of their own private pursuits (like commercialisation) [46, 47]. This can be explained by different sets of values or cultures on display by public and private institutions [30], just as the public good (as we have argued) does not always mean the same thing to an ethicist as it does to an economist.

As we have defined it, the public good means that stakeholders may not agree ultimately on the goal (or fate) of research in terms of shared benefit and solidarity. In this vein, it was written about biobanks that ‘competing, but ultimately compatible, interests’ of multiple agents often find they share values ([48] p. 9). We would disagree: it might be better said that conflicts between public and private are not inexorable but are culturally engrained; compatibility should certainly not be assumed, and, in fact, scepticism seems warranted.^6^ The chief danger is a misconstrued narrative about the public good that conceals a reluctance to be critical of business approaches (or to be less than appreciative of public ones). This is, of course, also a comment on the role of private and state interests in innovation, and the controversial aspects of expanding or shrinking the role of government in innovation (e.g. basic science, research and development) that go beyond the focus of this paper [49].

Instead, our message is short: one should challenge superficial explanations that hold to the benefits of markets without being critical of the processes—even injustices—that are involved [9]. In a culture that does not question such assumptions, there are clear trade-offs, as Olson speculates, ‘Perhaps science has assimilated the mores of the “new economy” a bit too readily’ ([20], p. 941). We have given examples of the achievements possible when not driven by financial gain; these accomplishments challenge the assumptions about public inefficiencies. Regardless, these assumptions continue to be carefully and advantageously communicated to convince others that innovation is driven by profit. In reality, the public infrastructure is not only essential, but ultimately able to compete, accelerate, and achieve.

In this regard, the rhetoric of hype or hope may be driven by questionable short-term gains (such as a patient’s vulnerablity for a last chance cure), but one should also question the significance of vested interests when a new, expensive drug is marketed to a deliberately impoverished health system. These must be challenged in the context of the long-term damages to socio-political elements rooted in the public sphere.

We caution, therefore, the erosion of the public good that produces, conserves, and preserves resources for current and future generations. The public good may substantiate ideas such as ‘open science’—one scheme in this regard is presented by Maynard Olson in this issue of *Human Genomics*, in which ‘investigators and small laboratories tap directly into a truly communal resource’ as an alternative to the tendency to build (and the connotations of) research behemoths. Our model of genomic solidarity supports this sentiment, although giant infrastructures can be welcomed, and to a degree, are inescapable when studying large publics because ‘communal’ is defined as providing information to publics but also inviting them to take part *en masse* as *individually informed* participants. A public bad leaves scope for secrecy or hype-oriented misinformation; and is bound to discourage participation, and instead the public become *subjects*. The public good, then, creates space for engagement and information dissemination; and requires an obligation for truthful claims, honest brokering and research integrity. Researchers in both big and more modest institutions equally are bound by the same solidarity. In this respect, if these giants fall—the active structures that support public science—then so would its contributions to the public commons and scientific method. Instead, researchers focused on the shared ideals of Merton’s principles of transparency, objectivity, disinterest and scepticism, will be forced into the private vision of ‘interested enquiry’ and ‘secret knowledge’ [14]. Merton’s principles are still fundamental to the way in which the dialogue between the science complex and society occurs, and they are necessary to ensure that research programmes evolve in a way that societal needs, expectations and concerns can be addressed, and benefits can be produced. It is likely that in market-obsessed environs, scientific integrity becomes eroded. Moreover, market priorities particularly affect the ways in which research endeavours are disseminated to colleagues and publics [50]. We therefore challenge prevailing neoliberal ideas and suggest that continuation of these strategies may evermore rely on hype and hope. Ultimately, that likely will undermines public infrastructure and, inevitably, the cost will be public trust.

## Conclusion

While legalisation has tended to dominate social commentaries about CRISPR, we have explored the role of other ‘giants’ as key players in the innovation of gene editing. In so doing, we have examined the biomedical research complex comprising individuals, institutions and cultures. Although multiple disassociated visions of social, legal and fiscal reality thrive within this complex, the question for us has been whether these separate visions of discovery evoke one ethical paradigm that outclasses another. We do not conceive of our world as one in which markets should be entrusted with making important decisions. Rather, we acknowledge the extent to which market players remain fundamentally dependent on public infrastructures and previous efforts made by (often anonymous, often public) others. An enquiry is urgently required to see whether forgoing these contributions through marketisation could in fact stifle or already has stifled progress.

The current world is one in which both public and private players have their place, and their supporters and detractors. If there is such a thing as an ideal community, there is unlikely to be a single guiding vision of innovation. However, careful study of the specific incremental contributions of individuals, institutions and networks within science, and how social and economic ideals affect them, will allow us to articulate the values that lead to truly prosperous science—not just economic gains, but also the augmentation of discoveries that may fundamentally change peoples’ lives. CRISPR is already a technical ‘disruptor’ [51]. We should consider now how its potential should be turned into a ‘health disruptor’. Otherwise, it may become another an unrealised promise, another invention encumbered by hype and hope. In this respect, public interest rhetoric is entangled with market agendas and, arguably, still dominates the ways in which many parties think about doing successful research—yet, the examples of UK Biobank and the IHGSC, used here, should be a warning to those acquiescent to that stilted dogma.

In the end, the single, major giant that supports biomedical sciences, including genomics, is ‘the public’ or, realistically, a constellation of all publics. We have articulated the concept of the public good as a solidary, a community that finds it worthy to protect the shared interests of research. This is what HUGO meant when it articulated ‘genomic solidarity’, with the public and scientists as joint owners in discovery and opportunity [29]. If genomic research becomes infused with the public good—one in which participants and scientists stand together for a common purpose that benefits humanity—then that is something to nurture to provide a viable and sustainable alternative to purely commercial research.

Here, our aim has been to open up avenues for deliberation for progress in genomic research. What is required now is a differential investigation into the roles of public and private efforts to create and translate basic science into public benefits via a ethical framework that does not instigate hype and hope as a ‘tried and trusted’ mechanism to drive innovation. There are a number of challenges for translating genomics for the public good, which can be brought together under three headings:

1. Conceptual—How can these challenges be practically framed within a conception of the public good?
2. Scientific—How can this framework create trust, promote progress and encourage investment in science?
3. Political and social—How can different agents (private and public) work within, and promote the goals of, this framework?

It is important, however, not only to investigate these issues and how they can be addressed but also to examine the indicators of scientific success (metrics) and the mechanisms that best reward fruitful research. The concept of genomic solidarity allows us to measure the extent to which genomics is or can be harnessed for the public good, so that both the public and the scientists share the benefits and opportunities.

## Footnotes

1 The first human draft genome cost at ‘least $500 million’, and a genome sequence in 2016 was below $1000); see https://www.genome.gov/sequencingcosts/

2 Heath’s reference is to the term ‘catallaxy’ used by Friedrich Hayek (via Ludwig von Mises) to describe spontaneous market order that would come about between different economies. In effect, Hayek believed that trade is fundamentally a pattern of mutually beneficial interactions

3 Paraphrasing from Umberto Eco’s ‘Dicebat Bernardus Carnotensis’, foreword in: [5] p. pxiii

4 See https://www.genome.gov/sequencingcosts/

5 Capture is the tendency for ‘private interests’ to appropriate public goods for their market value. It explains how market disinterest in worthless goods becomes a newfound interest, often by way of a spark that ignites a flurry of profiteering hubbub. A public good suddenly becomes a marketisation imperative. It might be as simple as turning a public road into a toll road; or as complex as sequencing a (very long) reference genome and claiming that being the first to do so rightly transfers ownership of the ‘human genome’ [25]

6 Knoppers, the then Chair of the HUGO Ethics Committee, suggested that patents would not be incompatible with ‘common heritage’ as long as consent and contracts were valid. Below, we discuss one of the examples used by Knoppers to strengthen our difference to her claim, noting that in fact deCODE’s efforts, rather than justifying ‘moving beyond historical distrust’ of industry [26], became an example of why scepticism and suspicion are well placed

7
*UNESCO Universal Declaration on Bioethics and Human Rights* (2005): Article 13—solidarity and cooperation: ‘Solidarity among human beings and international cooperation towards that end are to be encouraged’

8 Section 3.8. The Biobank’s Access Procedures also offer up a remedy: if ‘UK Biobank considers that an Unreasonable Restriction exists or is likely to exist, then it shall promptly notify the Applicant, and automatically, on receipt of such notification, the Applicant shall be deemed to grant a perpetual, irrevocable, worldwide, fully paid-up, royalty-free, fully sub-licensable licence to UK Biobank to use such Applicant-Generated Invention in order to remove or mitigate the Unreasonable Restriction’; (s. B8) http://www.ukbiobank.ac.uk/wp-content/uploads/2011/11/Access_Procedures_Nov_2011.pdf

9 See Myriad case: cDNA could be patented, but not human genomic DNA in its natural form; *Assoc. for Molecular Pathology v. Myriad Genetics, Inc*. 569 U.S. (2013).
